# Our ideas for introduction of single-port surgery

**DOI:** 10.4103/0972-9941.72400

**Published:** 2011

**Authors:** Yutaka Kojima, Yuichi Tomiki, Kazuhiro Sakamoto

**Affiliations:** Department of Coloproctological Surgery, Juntendo University, Tokyo, Japan

**Keywords:** Single-incision access surgery, single port surgery

## Abstract

Single-port surgery, which is also called single-incision laparoscopic surgery (SILS), laparoscopic single-site surgery, or single-port access surgery, has been performed in several countries. However, it has not been widely adopted throughout the world because there still remain some challenging problems to be solved, in terms of safety and technology, and the majority of devices specific to SILS are under development and have not been approved by the Japanese Pharmaceutical Affairs Law. Herein, we introduce single-incision access using existing surgical devices that will give us the opportunity to adopt SILS to our hospital.

## DESIGN OF OUR DEVICE

**Figure 1:**
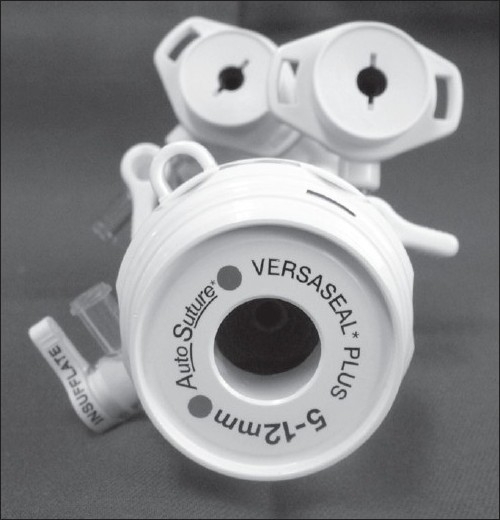
These ports were arranged in an inverted triangle.

**Figure 2:**
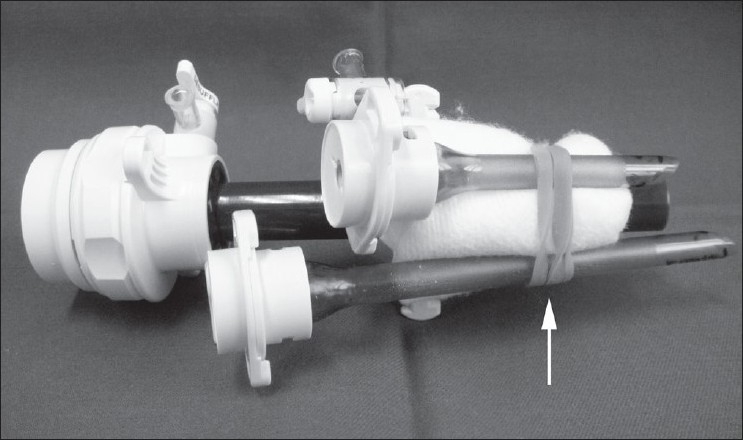
Wet absorbent gauze was then placed between the ports to maintain an airtight seal between them and to alleviate the interference with the ports, and the fulcrum was fixed with rubber (arrow). We fixed the long and short port to avoid interference with the port-head.

**Figure 3:**
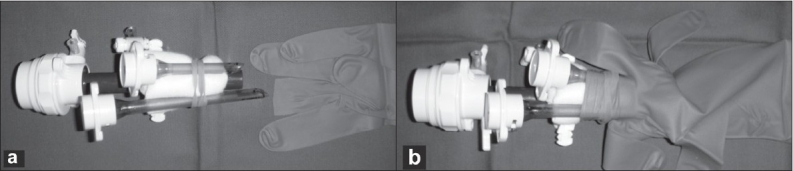
(a, b) The fixed port was inserted into the finger of a glove, which was removed with scissors, and the fulcrum was further fixed with rubber.

**Figure 4:**
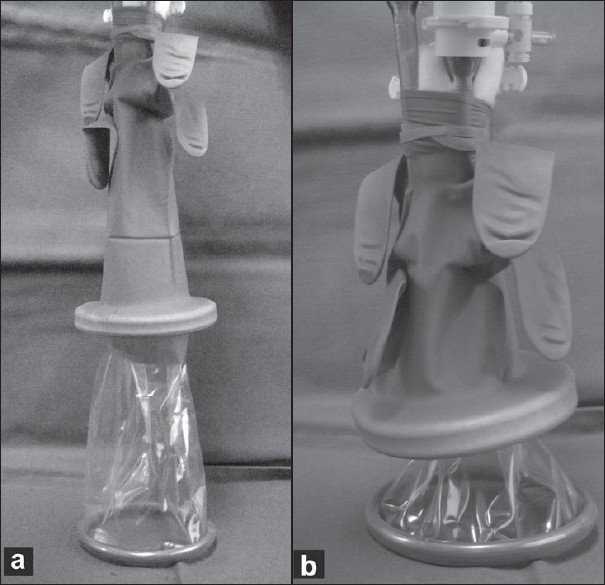
(a, b) The glove with the port was wrapped around a wound retractor

**Figure 5:**
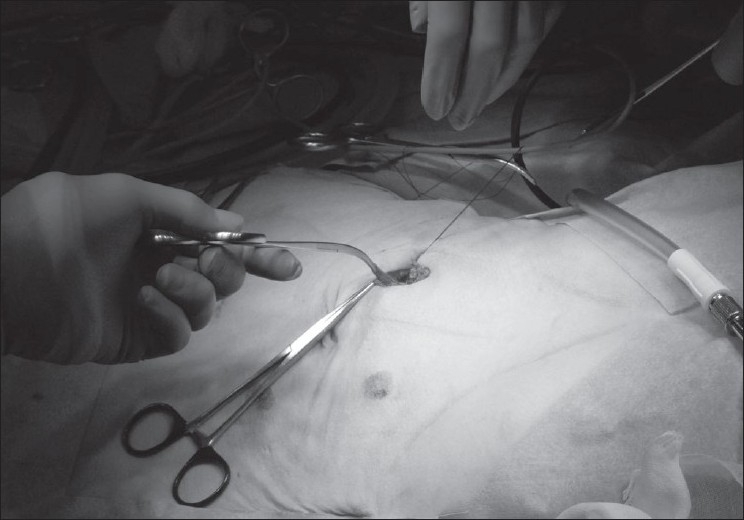
An approximately 2.5 cm incision was made in the stoma for cancer with significant infiltration to the surrounding tissues.

**Figure 6:**
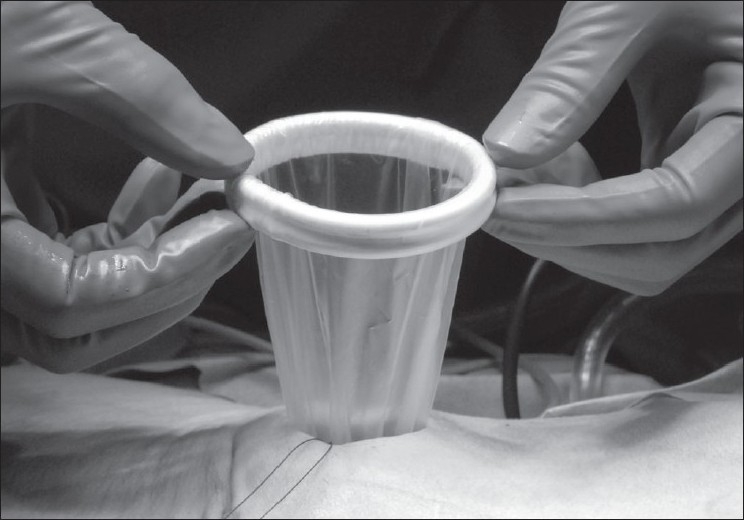
The wound retractor was placed into the incision.

**Figure 7:**
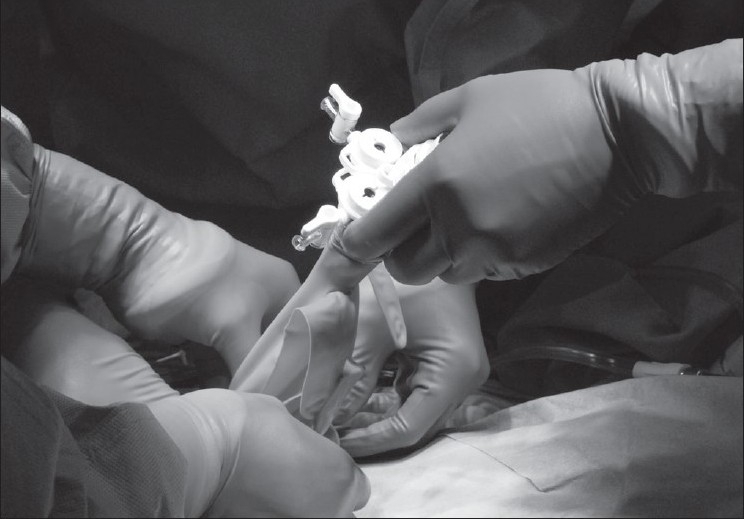
The glove with the port was wrapped around the wound retractor.

**Figure 8:**
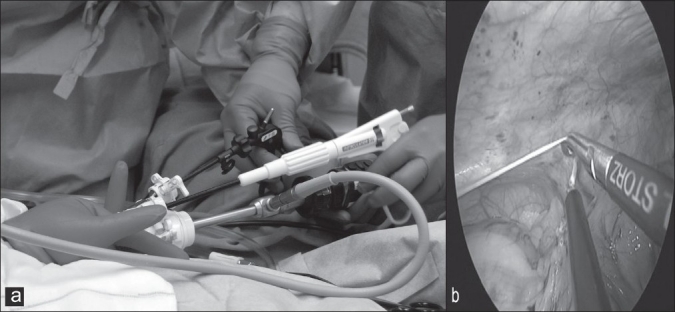
(a,b) There were no problems with the forceps procedure.

Three ports, BLUNT PORT PLUS^™^ (Covidien, Mansfield, MA, USA), SEPARATOR 5 × 100 mm (Applied Medical, Rancho Santa Margarita, CA, USA) and SEPARATOR^™^ 5 × 55 mm (Applied Medical, Rancho Santa Margarita, CA, USA), and ALEXIS^™^ wound retractor S size (Applied Medical, Rancho Santa Margarita, CA, USA), were used. The long and short types of a 5-mm port were used to avoid interference with the port head. These ports were arranged in an inverted triangle, as described in Figure 1. Wet absorbent gauze was then placed between the ports to maintain an airtight seal between them and to alleviate the interference with the ports, and the fulcrum was fixed with rubber [Figure 2]. The fixed ports were inserted into the middle finger of a glove, which was removed with scissors, and the fulcrum was further fixed with rubber [Figure 3a and b]. We used a surgical glove that was used in operation usually and size of the glove was 6 or 6.5. The glove with the ports was wrapped around a wound retractor, as described in Figure 4a and b. We adjusted amount of gauze among the ports to part of fulcrum of the ports into wound size. So the glove and ports were fixed around the wound. An approximately 2.5 cm incision [Figure 5] was made in the stoma for sigmoid cancer and rectal cancer with significant infiltration to the surrounding tissues, and the wound retractor was placed into the incision [Figure 6]. The glove with the port was wrapped around the wound retractor [Figure 7] and used at the site to observe the peritoneal cavity and to ablate. There were no problems with the forceps procedure [Figure 8a and b].

## DISCUSSION

Various devices are used in single-port surgery, including SILS port™ (Covidien), Uni-X™,[1] and R-port™,[2] but they are only allowed for use in limited facilities. Also, the instrumentation may translate into increased costs. We performed single port surgery using the existing surgical devices. The 5-mm long type and short type ports decreased the interference with the port head. Furthermore, the airtight seal was maintained by placing wet absorbent gauze between the ports. The fixation of the glove with the port into the wound retractor maintained the airtight seal and afforded excellent mobility. There is a possibility that we have difficulty of operability which has been described as a real disadvantage of single port surgery. At that time, we should not hesitate to convert to procedure of standard multiport laparoscopic or open surgery. Merchant *et al*.[3] also reported their cases of cholecystectomy, hemicolectomy, gastrectomy, and oesophagectomy using Gelport. Similar to laparoscopic surgery, which has been widely used as a standard procedure, it is expected that single port surgery will be used worldwide. The procedure that we introduced here using existing surgical devices may be readily available, cost-effective, and useful.
